# A novel recyclable furoic acid-assisted pretreatment for sugarcane bagasse biorefinery in co-production of xylooligosaccharides and glucose

**DOI:** 10.1186/s13068-021-01884-3

**Published:** 2021-02-02

**Authors:** Lin Dai, Tian Huang, Kankan Jiang, Xin Zhou, Yong Xu

**Affiliations:** 1 Jiangsu Co-Innovation Center of Efficient Processing and Utilization of Forest Resources, College of Chemical Engineering, Nanjing Forestry University, No. 159 Longpan Road, Nanjing, 210037 People’s Republic of China; 2 Key Laboratory of Forestry Genetics & Biotechnology (Nanjing Forestry University), Ministry of Education, Nanjing, 210037 People’s Republic of China; 3 Jiangsu Province Key Laboratory of Green Biomass-Based Fuels and Chemicals, Nanjing, 210037 People’s Republic of China; 4 School of Basic Medical Sciences and Forensic Medicine, Hangzhou Medical College, Hangzhou, 310053 People’s Republic of China

**Keywords:** Furoic acid, Recyclable, Xylooligosaccharides, Sugarcane bagasse, Enzymatic hydrolysis

## Abstract

**Background:**

Pretreatment is the key step for utilizing lignocellulosic biomass, which can extract cellulose from lignin and disrupt its recalcitrant crystalline structure to allow much more effective enzymatic hydrolysis; and organic acids pretreatment with dual benefic for generating xylooligosaccharides and boosting enzymatic hydrolysis has been widely used in adding values to lignocellulose materials. In this work, furoic acid, a novel recyclable organic acid as catalyst, was employed to pretreat sugarcane bagasse to recover the xylooligosaccharides fraction from hemicellulose and boost the subsequent cellulose saccharification.

**Results:**

The FA-assisted hydrolysis of sugarcane bagasse using 3% furoic acid at 170 °C for 15 min resulted in the highest xylooligosaccharides yield of 45.6%; subsequently, 83.1 g/L of glucose was harvested by a fed-batch operation with a solid loading of 15%. Overall, a total of 120 g of xylooligosaccharides and 335 g glucose could be collected from 1000 g sugarcane bagasse starting from the furoic acid pretreatment. Furthermore, furoic acid can be easily recovered by cooling crystallization.

**Conclusion:**

This work put forward a novel furoic acid pretreatment method to convert sugarcane bagasse into xylooligosaccharides and glucose, which provides a strategy that the sugar and nutraceutical industries can be used to reduce the production cost. The developed process showed that the yields of xylooligosaccharides and byproducts were controllable by shortening the reaction time; meanwhile, the recyclability of furoic acid also can potentially reduce the pretreatment cost and potentially replace the traditional mineral acids pretreatment.
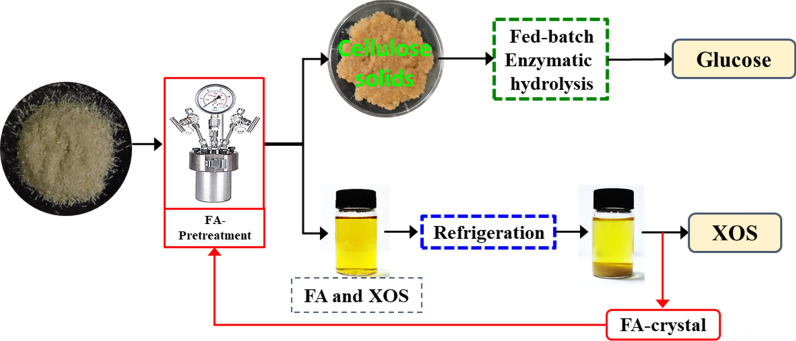

## Background

Increasing demands for chemicals and energy have driven the shift toward the exploration of alternative resources; therefore low-cost and renewable lignocellulosic materials are stimulating researchers to develop techniques and methods that can convert these resources into high value-added products [1–3]. Sugarcane bagasse (SCB), a type of fibrous residues, can be obtained from the juice extracted from sugarcane during the sugar production process [4]. Like other fibrous residues, SCB contains high amounts of polysaccharides, especially for cellulose and hemicellulose [5, 6]. Because of its lower ash content, the feedstock SCB has numerous advantages over other agro-based residues (such as corn stover and wheat straw); thus, it is a preferred candidate for biorefinery [7].

However, the native structure of SCB, like other lignocellulosic biomasses, is strongly recalcitrant to enzymatic hydrolysis. For this reason, the implementation of the pretreatment that can deconstruct the lignocellulosic entanglement is necessary [8–10]. Thus, pretreatment is considered as the key step to deconstruct the complex structure of lignocellulose [11]. According to previous studies, acidic pretreatments, including mineral and organic acids, have been proven to effectively disrupt the recalcitrant crystalline structure of cellulose and to efficiently enhance its enzymatic hydrolysis [12, 13]. Organic acid pretreatments are more desirable compared to dilute mineral acid pretreatments because of its many benefits in enzymatic hydrolysis, including generating lower amounts of degradation products and ability to directly generate value-added products like xylooligosaccharides (XOS) [14–16]. XOS are particularly attractive products because of their potential function in enhancing human immunity and promoting the growth of bifidobacteria and lactobacillus [17–19]. Concurrent production of XOS and fermentable sugars can effectively improve economic efficiency of SCB biorefinery [20]. However, the separation of acid catalyst and the recovery of product after the acid pretreatment are still challenging due to not only the technical issues, but also the economic problems. Therefore, it is urgent to speed up the technological innovation that can help augment the utilization and processing of SCB resources.

Furoic acid (FA) as a recyclable organic acid was first employed for producing XOS and pretreating SCB material. FA is a heterocyclic carboxylic acid, consisting of a five-membered aromatic ring and a carboxylic acid group and has been widely used in food products as preservative and flavoring agent; its solubility is only 37 g/L in 25 °C water and it is easily recovered by cooling and natural crystallization [21]. In the present study, we designed an integrated biorefinery process, which using FA as catalyst for xylan degradation and as a pretreatment agent—a part of the attempts to develop a new biorefinery process, starting from FA pretreatment.

## Results and discussion

### Influence of FA concentration, and hydrolysis temperature and time on conversion of hemicelluloses

**Fig. 1:**
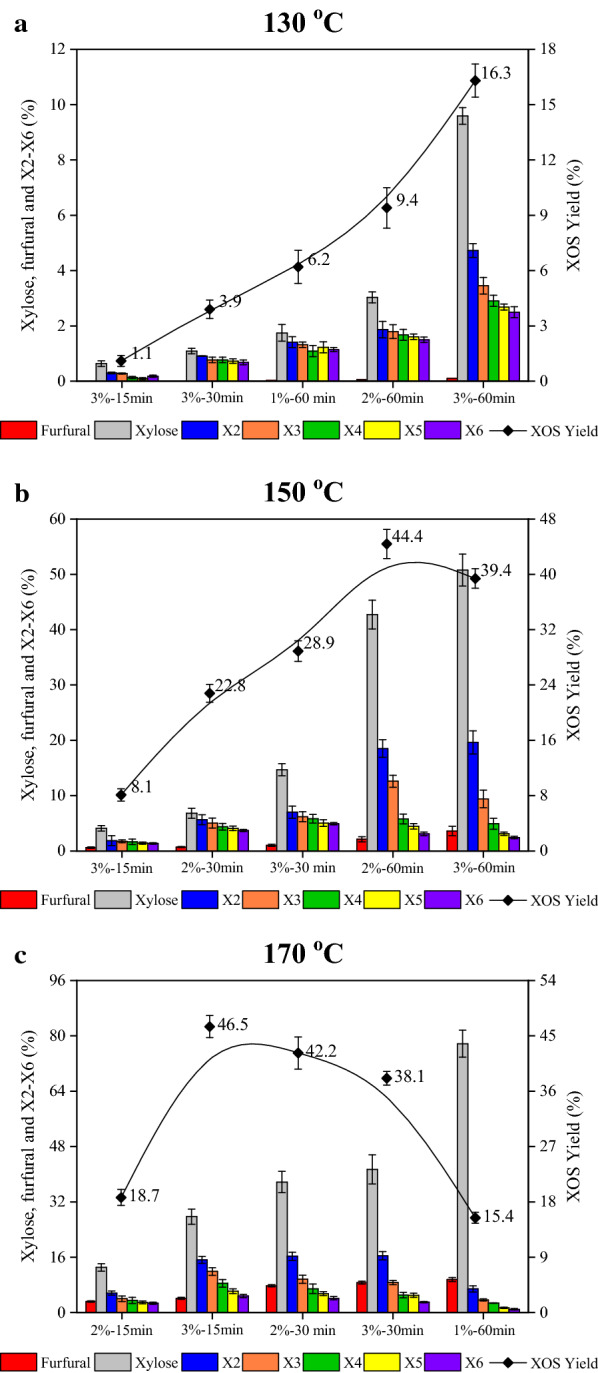
Yields of furfural, xylose, X2–X6, and XOS in hydrolysate of SCB pretreated with different concentrations of FA for varying durations at different temperatures: **a** 130 °C; **b** 150 °C; and **c** 170 °C

In an acid-hydrolysis process, xylan was first degraded into polysaccharides with high degree of polymerization (DP); and these polysaccharides would be further degraded into oligomers with low DP or xylose [14]. As a result, in the presence of acid catalyst, the XOS and xylose yields increased gradually with increasing reaction temperature and hydrolysis time. However, as retention time was further prolonged, XOS also could be degraded into xylose and furfural. Therefore, a suitable reaction condition is required so as to achieve the highest XOS yield. Three main parameters that affect both the degradation rate and the selectivity, including FA concentration (1–3%), hydrolysis time (15–60 min) and hydrolysis temperature (130–170 °C), were investigated [7], and the results, presented as yields of furfural, xylose and XOS with a DP range of 2–6, are displayed in Fig. 1a–c.

As can be observed in Fig. 1, both the hydrolysis temperature and hydrolysis time significantly affected the XOS yield. At relatively low reaction temperature and short hydrolysis time, the XOS yield was very low: the XOS yield produced by the use of 3% FA for 15 min at 130 °C was only 1.09%. It can also be seen that the XOS yield was significantly enhanced when the hydrolysis time was increased from 15 to 60 min: the XOS yield generated by the use of 3% FA for 60 min was 16.3%. According to the chromatogram, some xylo-saccharides with sizes of larger than X6 could not be degraded into XOS at a low temperature. Conversely, xylan was easy to be hydrolyzed into soluble polymers with lower molecular weights at high temperature. Figure 1a and b show that the XOS yield rapidly increased from 3.9% (130 °C, 3% FA, 30 min) to 28.9% (150 °C, 3% FA, 30 min); similar observation was also observed when the hydrolysis temperatures were 150 °C and 170 °C, in which the XOS yield was rapidly increased from 8.1% (150 °C, 3% FA, 15 min) to 46.5% (170 °C, 3% FA, 15 min). In addition, too long hydrolysis time resulted in a markedly decline in XOS content, which could be ascribed to the further degradation of some XOS under such harsh reaction conditions. Thus, we observed that XOS yield at both 150 and 170 °C first increased and then decreased, and with prolonged hydrolysis time and higher temperature, higher amounts of X2 and X3 and lower amounts of X5 and X6 were obtained. Using 3% FA as the catalyst, a remarkable decrease in XOS yield at 170 °C from 46.5% (15 min) to 38.1% (60 min) was observed; and accordingly, a rapid increase of xylose and furfural yields was observed.

Taken together, in the case of the low reaction temperature, it required a relatively long hydrolysis time and it is economically unfavorable. However, longer hydrolysis time resulted in further hydrolysis of oligosaccharides into smaller molecules byproducts, xylose and furfural. Although similar XOS yields (44.4 versus 46.5%) were obtained at 150 and 170 °C at 60 and 15 min, it could be observed that the yields of xylose and furfural with a hydrolysis time of 60 min were significantly higher than that of 15 min. Apparently, the longer reaction time resulted in higher by-products (xylose and furfural) yields. These results indicate that the production of XOS and byproducts in the FA-assisted hydrolysis process can be controlled by changing FA concentration, hydrolysis temperature and reaction time. It has been accepted that a high reaction temperature with short hydrolysis time is much more conducive to the formation of oligosaccharides with lower by-product yields. Optimization of the assays showed that the highest content of XOS generated (170 °C for 15 min with 3% FA) was 11.9 g/L (3.77 g/L X2, 3.04 g/L X3, 2.45 g/L X4, 1.54 g/L X5 and 1.11 g/L X6) with a yield of 46.5%

### Furoic acid recovery

Nowadays, green and sustainable development, which aims to balance the environment/resource and economic growth, is a matter of significant importance facing all countries. Thus, a required appeared to re-design the processes for preventing hazardous chemical syntheses, minimizing wastes and increasing efficiency. The use of stronger maleic acid (H_2_SO_4_ or HCl) for pretreating SCB might result in lower catalyst loading and short reaction time, but it is not suitable for recycling and repeated extractions because of the environmentally hazardous and costly [13]. In contrast, FA is a heterocyclic monocarboxylic acid with a low water-solubility, thus rendering it can be easily extracted and recovered for further reuse, mostly by using the natural crystallization method of cooling the acid liquor.

**Fig. 2:**
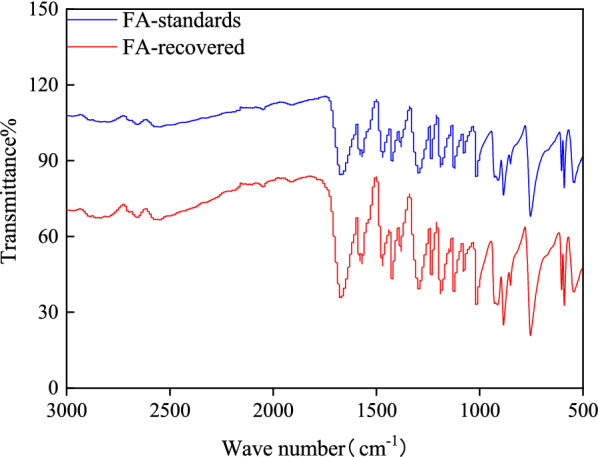
FTIR spectra of standard and recovered furoic acid

After being separated by filtration, the acid liquor was triple-concentrated through rotary vacuum evaporation, and the concentrated hydrolysate was then refrigerated overnight at 4 °C, then 95% FA crystallized and gradually precipitated. In order to determine whether the acid remained unaffected, HPLC analysis was initially used to determine the change of the FA. In addition, FTIR was also applied to analyze the FA crystals and to compare them with the crystals of the FA standards. The HPLC results confirmed that the peak of the collected crystals was the same as that of the standard and the content of FA after hydrolysis showed almost no lose. As shown in Fig. 2, the FTIR spectra showed that there were no significant differences between the crystals of FA and those of the standard [22]. The results of both HPLC and FTIR analyses confirmed both the identity and the purity of FA, indicating that the recovered FA was identical to the FA standards, thus it may suitably be recycled in additional hydrolysis rounds.

### Enzymatic hydrolysis of pretreated solid residues

**Fig. 3:**
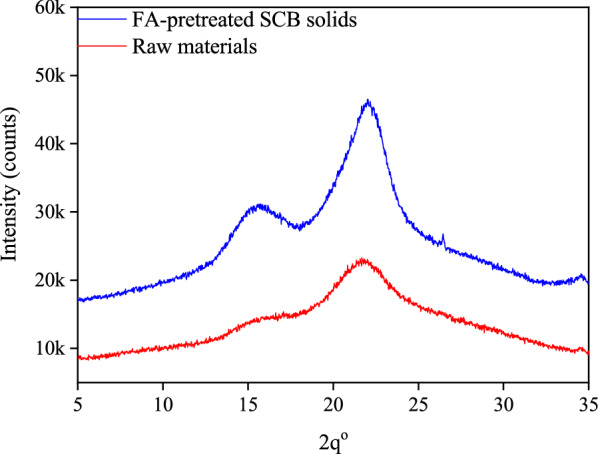
XRD patterns obtained from the un-pretreated raw material and FA-pretreated SCB solids

**Fig. 4:**
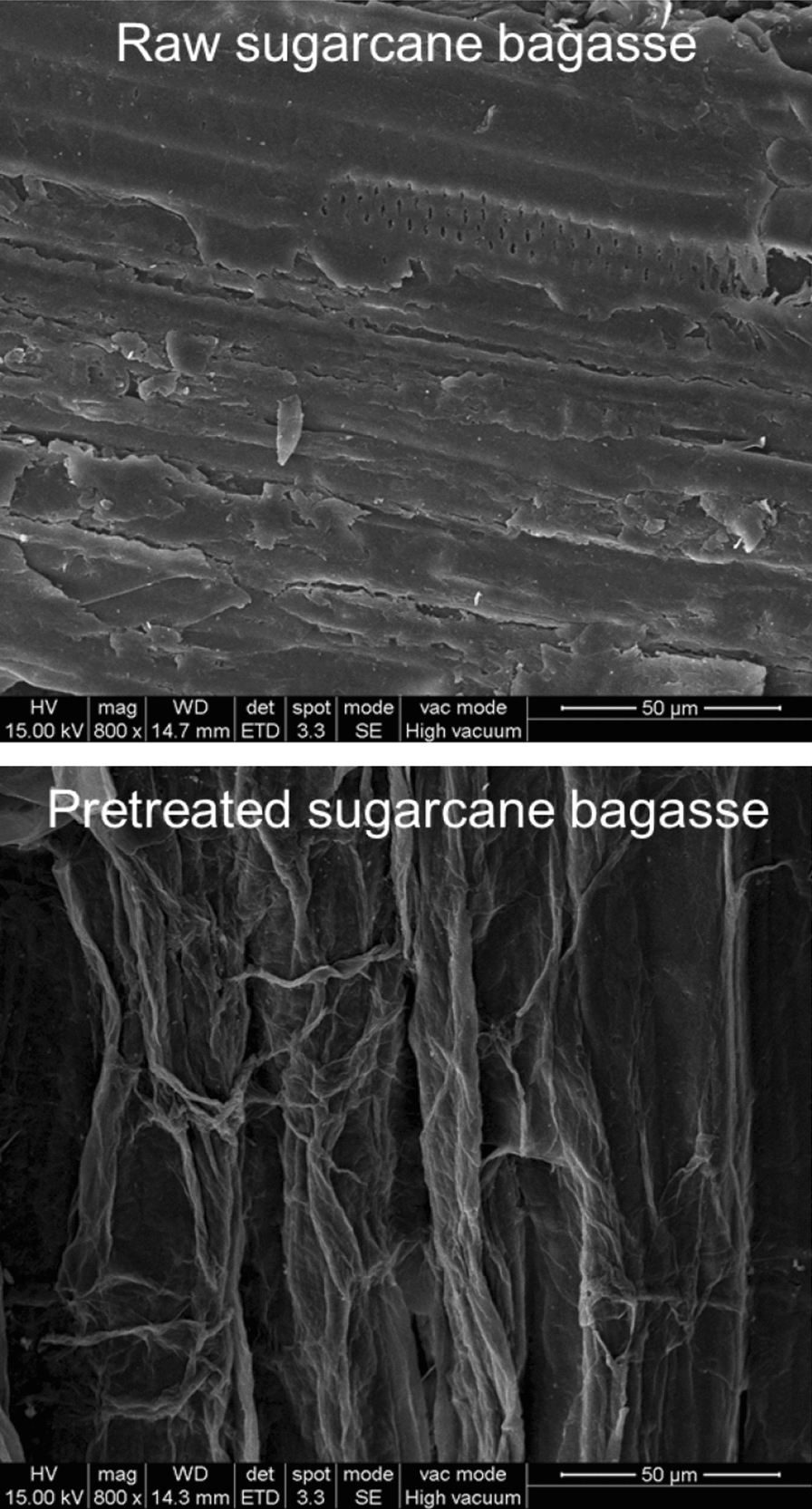
Scanning electron micrographs (SEM) of pretreated and untreated sugarcane bagasse solid

It is known that crystallinity of cellulose can highly impact its enzymatic digestibility. XRD analysis (Fig. 3) showed that the characteristic peak of cellulose I at 2*θ* = 21.9° were observed in both the un-pretreated biomass and the FA-pretreated biomass. The CrI, which is the ratio between the crystalline portion in cellulose to the amorphous portion, of the FA-pretreated sample (48.8%) increased compared to that of the un-pretreated sample (34.3%). The increase of CrI is mainly attributed to get rid of amorphous hemicellulose. The removal of the xylan-riched hemicellulose and lignin can break the entanglement of cellulose, hemicellulose and lignin, and improve the accessibility of cellulases to cellulose. The XRD results indicated that the pretreatment with FA could increase the cellulose portion availability of SCB, suggesting that it can increase the cellulolytic digestibility of SCB in the same manner as the other pretreatment methods [23, 24]. Furthermore, the SEM (Fig. 4) images showed that the surface of un-pretreated raw SCB is smooth and compact. After FA pretreatment, the surfaces of pretreated SCB appeared rough and etched, and seem to feature much more newly exposed surfaces. Larger exposed surface areas and more micropore quantities that occurred in FA-pretreated SCB offer more probability for the action of cellulases [25]. Altogether, FA-assisted prehydrolysis is a feasible and promising pretreatment method for further processing SCB.

After the pretreatment with 3% FA, 85% of xylan was removed from SCB, while XOS was directly converted into a liquid phase, causing the content of glucan in the pretreated solid residues to increase from 42.7 to 62.1%; and most of the glucan (88.6%) was reserved. The reserved glucan can usually be degraded into glucose, which is easy to be transformed into biofuels or other biochemical compounds; however, it is well known that the activity of cellulases will be inhibited by inhibitors from degradation, such as formic acid, furfural, and HMF, as well as by lignin derivatives (phenolic compounds). Thus, prior to the enzymatic hydrolysis process, the pretreated solids were firstly rinsed with water to get rid of the inhibitors [26]. Moreover, increasing the solids loading not only can enhance the final fermentable glucose concentration, but also reduce the overall production cost by reducing the equipment size, the associated energy consumption, and the burdens of the downstream processing. Therefore, a higher solid loading is preferred in process of enzymatic hydrolysis; and based on that, batch and fed-batch hydrolysis using varying solid loadings (5, 10, 15, and 20% w/v) to produce high-concentration glucose was conducted. In this assay, the Cellic CTec2 enzyme at a dose of 20 FPIU/g glucan was used; this enzyme contains a certain amount of β-glucosidase; therefore, β-glucosidase was not additionally added into the assay.

**Fig. 5:**
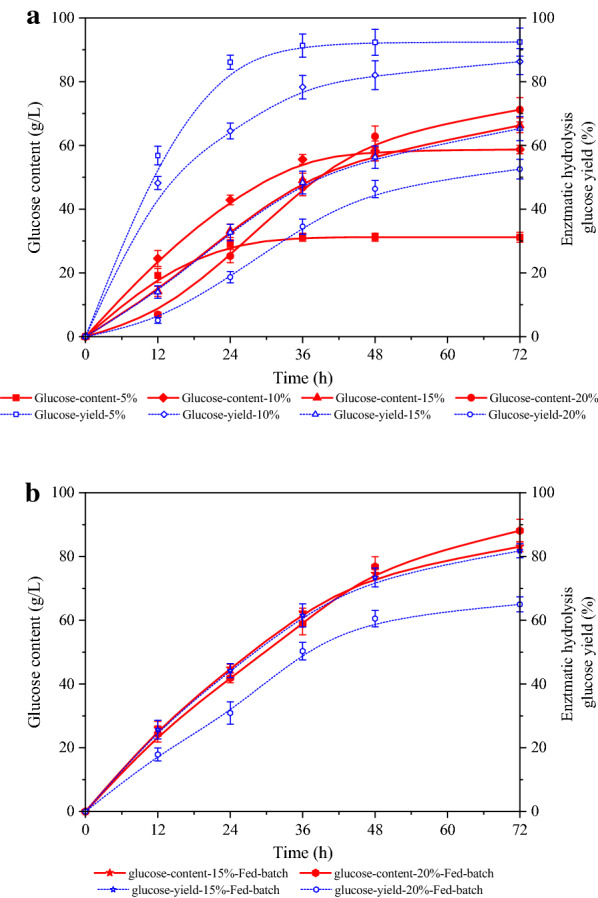
Performance of **a** batch enzymatic hydrolysis using 5–20% solid loadings; and **b** fed-batch enzymatic hydrolysis using 15 and 20% solid loadings

The course of glucose production depicted in Fig. 5a showed that the solid loading had a direct link with the glucose concentration in each enzymatic hydrolysate. After 96 h of reaction, glucose at concentrations of 31.2, 58.7, 66.3, and 71.2 g/L was released from the reaction with solid loadings of 5, 10, 15 and 20%, respectively. However, as can be seen, the solid in the system containing 5–10% solid loadings was liquefied within 6–12 h, and the relationship between the glucose concentration and the solid loading was nearly linear. On the other hand, the time of liquefaction of the system with the solid loadings of 15–20% was retarded to over 12 h, and the hydrolysis rate was also very slow. Yields of 90.8, 90.2, 74.2, and 63.1% were obtained from the system with the solid loadings of 5, 10, 15, and 20%, respectively. It can also be observed that the hydrolysis rate was high in the first 12 h; this could be explained by the reduction of the crystallinity and the increase of the available catalytic sites [27]. It is apparent that high hydrolysis consistency may result in difficulties in stirring the material due to high viscosity as a result of high solid loadings and the lack of free water in the system, which can limit the mass transfer; these events lead to product accumulation [28, 29].

Higher solid loadings can therefore lead to the decrease in glucose yield. When the solid loading was over 15%, there was a considerable amount of cellulose in the pretreated residue that was not hydrolyzed. Therefore, it could be concluded that 10% solid loading was the initial loading optimal for batch operation, according to the change of glucose concentration and the time of liquefaction. However, the glucose concentration was only 57 g/L when the solid loading was 10%. High ethanol concentration (> 4%, v/v) is one of the prerequisites that enable the industrial-scale ethanol distillation to be economically viable. Thus, it is necessary that glucose obtained from the enzymatic hydrolysis should over 80 g/L; this also indicates that the loading of the solid biomass that usually contains 40%-60 glucan should be over 15% to sufficiently achieve the available fermentable sugars [30]. However, the slurry of the fibrous material with high solid loadings has high apparent viscosity, which can result in poor mixing, as well as poor mass and enzyme distribution and poor heat transfer, causing the reduce of the enzymatic efficiency. One approach that can minimize these negative effects is by conducting fed-batch enzymatic hydrolysis [30].

Thus, we carried out fed-batch enzymatic hydrolysis of FA-pretreated SCB to produce glucose with high-concentration. As presented in Fig. 5b, 83.1 and 88.1 g/L glucose could be obtained, respectively, with fed-batch enzymatic hydrolysis of 15 and 20% solids loading. Apparently, at the same solid loading of 15%, hydrolysis time required for the fed-batch mode was lower than that required for the batch mode. At the fed-batch solid loading mode, although the glucose conversion decreased in both cases, the decrease in fed-batch mode was lower than that in batch mode, which still demonstrates that the fed-batch mode is an excellent way to produce high-concentration fermentable sugars. Altogether, the fed-batch hydrolysis could weaken the negative effects such as low free water content, poor mixing, and product inhibition, thus could enable the production of high glucose concentrations at high solid loadings. In addition, 88.1 g/L glucose could be obtained from 20% solids loading with fed-batch operation, but at a lower yield of 65.1%. Taken together, a fed-batch enzymatic hydrolysis of FA-pretreated SCB with 15% solid loading within 72 h is preferable for producing high-concentration fermentable glucose.

**Fig. 6:**
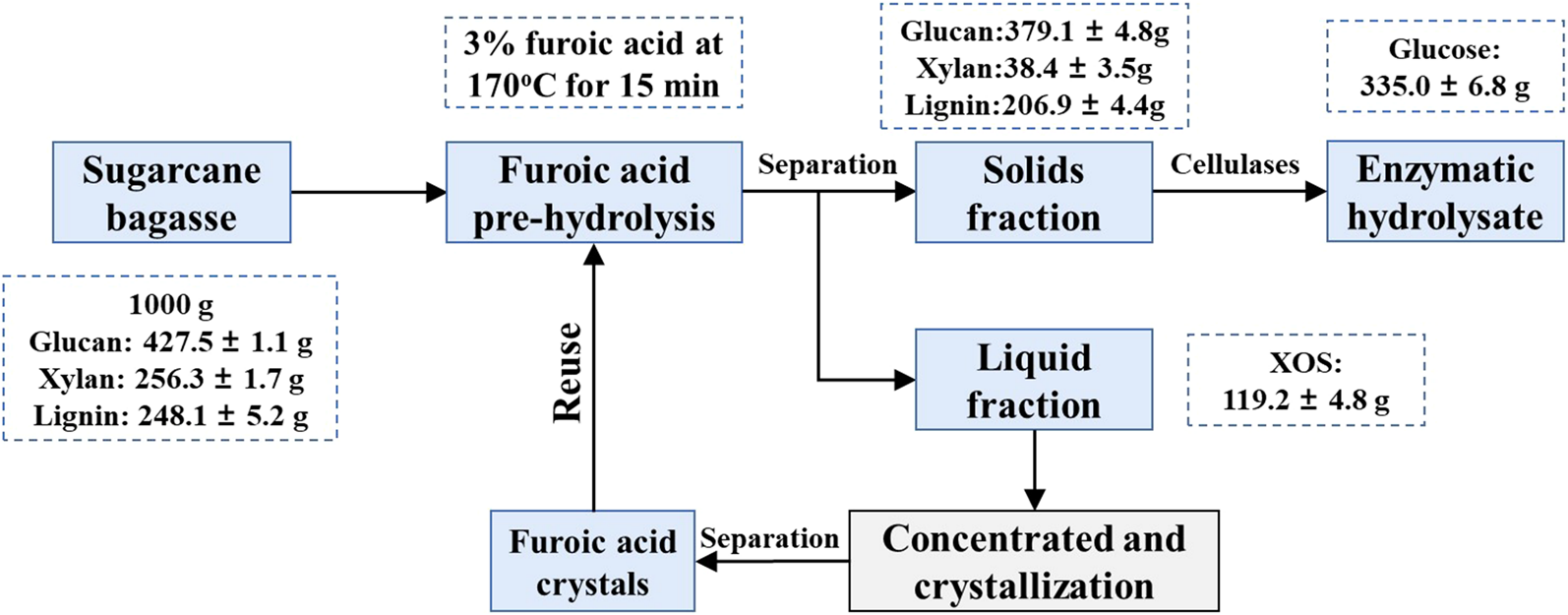
Mass balance for sugarcane bagasse biorefinery

Finally, the mass balances of the integrated process for XOS and glucose production were systematically calculated, as depicted in Fig. 6, approximately 120 g XOS and 335 g glucose products could be collected from 1000 g oven-dried SCB (containing 427 g glucan and 256 g xylan) starting from FA pretreatment. In aggregate, the recovery rates of xylan and glucan were 46.5 and 71.3%, respectively. The experimental findings suggest that FA pretreatment could be a promising and profitable option for the concurrent maximization of the economic value of SCB.

## Conclusion

In this study, a novel FA pretreatment method that can be applied in XOS and glucose production was developed. The optimal pretreatment conditions, which resulted in the highest XOS yield of 46.5%, were 3% FA, 170 °C, and 15 min. The fed-batch enzymatic hydrolysis of FA-pretreated SCB could successfully produce high concentration of glucose, which is over 8%. Overall, a total of 120 g of XOS and 335 g glucose were collected from 1000 g SCB starting from recyclable FA pretreatment. It is a strategy that the sugar and nutraceutical industries can be used to reduce the production cost.

## Materials and methods

### Raw materials and chemical composition analysis

SCB was harvested in October 2019 from Hainan Province, China. The harvested SCB was air-dried, milled, and then passed through 60 meshes; the obtained ground SCB was used as feedstock for subsequent processes. The chemical composition (wt%, oven-dry-weight basis) of SCB was: glucan, 42.7%; xylan, 25.6%; lignin, 24.8%; ash, 2.06%. The chemical composition of SCB was analyzed by the two-step sulfuric acid hydrolysis method, which is supported by the National Renewable Energy Laboratory (NREL) [31].

### Hydrolysis of sugarcane bagasse by furoic acid

SCB (3.0 ± 0.03 g) was mixed with 30 mL of 1–3% FA solution and loaded in a 50-mL stainless steel tube reactor, which was then immersed in preheated oil baths at 130–170 °C for 15–60 min; after the hydrolysis reaction was completed, the reactor was immediately removed from the oil bath and cooled down to room temperature. The resultant solid and liquid fractions were separated by filtration; and the solids were then subjected to enzymatic hydrolysis process [32].

### Batch and fed-batch enzymatic hydrolysis

Prior to enzymatic hydrolysis, the solid residues from the FA pretreatment were washed with distilled water and then air-dried to a constant weight (the moisture content was 7.6%). Both batch and fed-batch enzymatic hydrolysis assays were conducted in a screw capped bottles (250 mL) shaken at 150 rpm at 50 °C for 72 h: each bottle contained 50 mL sodium citrate buffer (0.05 mol/L, pH 4.8), and a constant cellulase concentration of 20 FPIU/g glucan (Cellic CTec2, Novozymes, NA, Franklinton, USA) was added into enzymatic hydrolysis system according to different solid loadings (5, 10, 15, and 20%). After enzymatic hydrolysis, the slurry was subjected to centrifugation for collecting supernatant. Fed-batch enzymatic hydrolysis was conducted with 5% (w/v) of solids (2.5 g dry weight) and enzymes; the two components (solids and cellulase) were simultaneously added into the hydrolysis reaction system every 6 h at the desired loadings [28, 30].

### Recovery of furoic acid from the hydrolysate

After being separated by filtration, the acid liquor was triple-concentrated by rotary vacuum evaporation (RV10, IKA, Germany), and the concentrated hydrolysate was then refrigerated overnight at 4 °C to allow FA to gradually crystallize and precipitate. The FA crystals were recovered by filtration through a glass fiber filter and were then compared with the crystals of the FA standard using Fourier transform infrared (FTIR) spectroscopy (Tensor 27-IR, Bruker, USA).

### Analysis of crystallinity of cellulose by X-ray diffraction

The crystallinity index (CrI) and the crystal size of cellulose from unpretreated and FA-pretreated SCB solids were analyzed by X-ray diffraction (XRD) at a voltage of 40 kV and a current of 40 mA. The data were collected from 5° to 50° at a 10°/min scanning rate. The CrI was calculated as the followed equation:

$${\mathbf{CrI}}\,{\mathbf{(\% )}} = \frac{{{\text{A}}_{{{\text{cryst}}}} }}{{{\text{A}}_{{{\text{cryst}}}} + {\text{A}}_{{{\text{amorph}}}} }} \times 100\% ,$$

where A_cryst_ is the area under curve of the crystalline cellulose and A_amorph_ is the area under curve of the amorphous cellulose [24].

### Analytical methods

Furfural and FA were analyzed by a high-performance liquid chromatograph (HPLC; Agilent 1260, USA) equipped with an Aminex Bio-Rad HPX-87H column (Bio-Rad Laboratories, USA); xylose, xylobiose (X2), xylotriose (X3), xylotetraose (X4), xylopentaose (X5), and xylohexaose (X6) were co-analyzed by a high-performance anion exchange chromatograph (Dionex ICS-3000, USA) coupled to a CarboPac™ PA200 column [33].

## Data Availability

All data generated or analyzed during this study are included in this published article.

## Abbreviations

SCB: Sugarcane bagasse
XOS: Xylooligosaccharides
XA: Xylonic acid
SB: Sugarcane bagasse
XA·Na: Sodium xylonite
DP: Degrees of polymerization
X2: Xylobiose
X3: Xylotriose
X4: Xylotetraose
X5: Xylopentaose
X6: Xylohexose
X7: Xyloheptaose
X8: Xylononylose
FTIR: Fourier transform infrared
CrI: Crystallinity index
XRD: X-ray diffraction
HPLC: High-performance liquid chromatography
HPAEC: High-performance anion exchange chromatography
